# Genomic Analysis of *Paenarthrobacter* sp. FR1 Reveals Its Marine-Adapted Pectin-Degrading System and Ecological Role in Carbon Cycling

**DOI:** 10.3390/microorganisms14010039

**Published:** 2025-12-23

**Authors:** Zulfira Anwar, Jixin Tao, Jing Lin, Yiran Cui, Hongcai Zhang, Xi Yu, Jiasong Fang, Junwei Cao

**Affiliations:** College of Oceanography and Ecological Science, Shanghai Ocean University, Shanghai 201306, China

**Keywords:** pectin degradation, carbohydrate-active enzyme (CAZyme) system, plant dead biomass, genus *Paenarthrobacter*

## Abstract

Microbial degradation of pectin is a fundamental process for the carbon cycle and a strategic approach for treating industrial residues. This study characterizes a novel marine bacterium, *Paenarthrobacter* sp. FR1, isolated from East China Sea intertidal sediment, which exhibits the ability to utilize pectin. Its draft genome (4.83 Mb, 62.92% GC content) is predicted to encode 4498 protein-coding genes. Genomic analysis revealed a rich repertoire of Carbohydrate-Active Enzymes (CAZymes) crucial for this process, including 108 glycoside hydrolases (GHs), 7 polysaccharide lyases (PLs), 35 carbohydrate esterases (CEs), and 11 auxiliary activities (AAs). Genomic analysis provides supportive evidence that FR1 may target both homogalacturonan (HG) and rhamnogalacturonan (RG) pectin domains, potentially through complementary hydrolytic and oxidative pathways. Phylogenomic analysis based on Average Nucleotide Identity (ANI, 83.56%) and digital DNA-DNA Hybridization (dDDH, 27.8%) confirmed its status as a potential novel species. Notably, FR1 is a rare *Paenarthrobacter* isolate with innate pectinolytic capability, a characteristic not previously documented in this genus. This strain’s unique enzymatic machinery highlights its importance in marine carbon cycling and provides a valuable biotechnological resource for degrading pectin-rich wastes.

## 1. Introduction

Pectin, a complex heteropolysaccharide primarily composed of α-1,4-linked galacturonic acid residues [1,2], constitutes a core structural component of plant cell walls and the middle lamella, accounting for 30–50% of the primary cell wall [3]. As one of the most abundant renewable biomaterials in terrestrial and marine ecosystems, pectin plays a pivotal role in the global carbon cycle [4]. Its recalcitrant branched structure, reinforced by methylesterification and rhamnogalacturonan cross-links, necessitates specialized enzymatic machinery for depolymerization. Marine environments receive substantial inputs of pectin from algal detritus and drifting plant matter, relying on microbial pectinolytic activity to convert this polysaccharide into soluble sugars [5,6]. This process sustains marine food webs and drives carbon and nitrogen fluxes [7]. Pectin hydrolysis is mediated by a suite of enzymes, including polygalacturonases (PGs), pectate lyases (PLs), and pectin methylesterases (PMEs), which act synergistically to depolymerize pectin into galacturonic acid monomers [8]. These enzymatic systems not only underpin the global carbon cycle but also possess significant biotechnological potential in areas such as biofuel production, food processing, and agricultural waste valorization [9].

The phylum Actinobacteria (Actinomycetota), renowned for its enzymatic diversity and adaptability to diverse ecosystems, has been extensively studied for lignocellulose degradation [10,11]; however, its pectinolytic potential remains underexplored. Bacteria within the genus *Paenarthrobacter* have gained attention for their metabolic versatility in degrading pesticides and aromatic pollutants [12,13]. Previous research has shown that the genus *Paenarthrobacter* is not entirely confined to terrestrial niches; several members, including strain FR1, have been isolated from marine environments, such as coastal waters and deep-sea sediments [14]. Metagenomic data obtained from these marine habitats further support the ecological relevance of this genus, indicating that *Paenarthrobacter* sequences are present in local microbial communities at measurable abundance [15,16]. But previous studies have not noted the involvement of *Paenarthrobacter* species in pectin catabolism, which remains a gap in understanding their ecological roles in the marine polysaccharide cycle. This study reports the genome of a novel marine strain, *Paenarthrobacter* sp. FR1, isolated from intertidal sediments of the East China Sea, which utilizes pectin as its sole carbon source. Genomic analysis revealed an integrated enzymatic arsenal for hydrolytic and oxidative pectin utilization pathways. The hydrolytic pathway includes polygalacturonase, rhamnogalacturonan lyase, and downstream galacturonic acid metabolism genes, while the oxidative pathway involves auxiliary activity (AA) enzymes. These findings establish *Paenarthrobacter* sp. FR1 as a novel type strain for investigating actinobacterial pectin metabolism in marine ecosystems and highlight its biotechnological potential for sustainable biomass conversion. This research expands the ecological repertoire of *Paenarthrobacter* beyond pollutant degradation, underscores its contribution to the marine carbon cycle, and provides genome-based insights for optimizing industrially relevant pectinase production.

## 2. Materials and Methods

### 2.1. Isolation and Identification of Strain FR1

To isolate intertidal bacteria that use pectin as their sole carbon source, intertidal sediment samples were collected from Chongming Dongtan Beach, East China Sea (31°26′ N, 121°57′ E), and 5 g of wet sediment was homogenized with 50 mL of sterile seawater for 15 min to separate microbial aggregates. After brief sedimentation, the supernatant was taken as the primary inoculum. Three consecutive enrichment culture cycles were carried out: 1 mL of the inoculum was transferred to 100 mL of artificial seawater medium (LMO: Na_2_HPO_4_ 6 GL^−1^, KH_2_PO_4_ 3 GL^−1^, NH_4_Cl 1 GL^−1^, MgSO_4_·7H_2_O 0.2 GL^−1^, CaCl_2_ 0.01 GL^−1^, NaCl 25 GL^−1^, trace element solution [17] 1 mL^−1^, vitamin solution [17] 1 mL^−1^, pH 7.2) supplemented with 0.2% (*w*/*v*) pectin (Macklin, Shanghai, China, Cat. No. 9000-69-5), and cultured at 28 °C and 150 rpm for 7 days; 5 mL of the first enrichment culture was transferred to fresh LMO artificial seawater medium supplemented with 0.5% pectin and cultured under the same conditions for 5 days; finally, 5 mL of the second enrichment culture was inoculated into LMO artificial seawater medium supplemented with 1.0% pectin for enhanced culture for 5 days. The enrichment culture was serially diluted and plated on LMO artificial seawater solid medium (15 GL^−1^ agar) supplemented with 0.5% pectin and 0.01% neutral red. After 5 days of incubation at 28 °C, colonies with a distinct hydrolysis halo were isolated. Pure cultures were obtained through three consecutive streaking purifications on 2216E solid marine medium.

### 2.2. Sole-Carbon-Source Cultivation Experiment and Crude Pectin Lyase Enzyme Activity Assay

The pectin hydrolysis ability of the purified strain was verified by evaluating its growth in a liquid LMO artificial seawater medium containing 0.5% pectin as the sole carbon source, with negative controls consisting of LMO medium without pectin and LMO medium containing pectin but without bacterial inoculation. While the solid medium was used for qualitative observation of colony formation, the liquid system enabled quantitative growth monitoring. To prevent thermal degradation, key medium components were sterilized separately: the pectin solution was autoclaved at 115 °C, while the trace element solution was sterilized by filtration. For the solid LMO-agar medium, cycloheximide (50 μg/mL) was added before solidification to inhibit fungal contamination. For liquid cultivation experiments, cells were harvested during the exponential growth phase by centrifugation at 4000 rpm for 10 min and washed three times with sterile artificial seawater. All cultivations in the sole-carbon-source medium were conducted in triplicate at 28 °C for 5 days, and bacterial growth in liquid culture was monitored by daily measurement of the optical density at 600 nm (OD_600_) using 200 μL samples. The crude enzyme activity of pectin lyase (PL) in the culture supernatant of *Paenarthrobacter* sp. FR1 was quantitatively analyzed using a commercially available Pectin Lyase Activity Assay Kit (Imagene, Beijing, China, Cat. No. SG102-1). The assay is based on the beta-elimination reaction catalyzed by PL, which cleaves the polygalacturonate backbone to produce Delta 4,5-unsaturated oligogalacturonates, which can be measured using their characteristic absorbance at 235 nm. Briefly, the FR1 strain was cultured in LMO liquid medium containing 0.5% percent pectin for 4 h at 28 °C; the culture was then centrifuged at 12,000 times g for 10 min (4 °C), and the resulting supernatant was collected as the crude enzyme solution. For the assay, 500 uL of 50 mM Glycine-NaOH buffer (pH 9.0) and 500 μL of the 1% (*w*/*v*) polygalacturonate substrate solution (prepared in pH 9.0 buffer) were mixed and pre-incubated at 50 °C for 5 min. The reaction was initiated by adding 100 μL of the pre-warmed crude enzyme solution, and the mixture was allowed to react for exactly 10 min at 50 °C. The reaction was terminated by adding 300 μL of 1 M HCl (hydrochloric acid). An inactivated enzyme control (enzyme added after HCl) and a blank control (sterile medium instead of enzyme) were run simultaneously. After centrifugation at 12,000 times g for 5 min, the absorbance of the supernatant was measured at 235 nm. One unit (U) of PL activity was defined as the amount of enzyme required to produce 1.0 micromole of Delta 4,5-unsaturated product per minute under the specified conditions (50 °C, pH 9.0), calculated using the change in absorbance (Delta A235), the total reaction volume, the enzyme volume, the reaction time, and the molar extinction coefficient (epsilon) of the product (5500 M^−1^ cm^−1^). All experiments were performed in triplicate and reported as the mean plus/minus standard deviation.

### 2.3. Whole Genome Sequencing and Assembly

Genomic DNA was extracted from bacterial cultures during the logarithmic growth phase using the Macklin™ Bacterial Genomic DNA Extraction Kit (Macklin, Shanghai, China) and strictly adhering to the manufacturer’s protocol. Draft genome sequencing was performed on the Illumina HiSeq 2000 platform with 2 × 150 bp paired-end (PE) libraries (458 bp insert size). Sequencing quality was systematically evaluated through per-cycle statistics, including base distribution, Phred scores, error rates, and positional bias analysis. Following quality control with fastp (version 0.20.0) [18], the clean data of *Paenarthrobacter* sp. FR1 yielded 8,505,114 paired-end reads, totaling 1.27 Gb of sequence data, providing a sequencing depth of 262.39×. Non-overlapping sliding window analyses (1–10 kb intervals) systematically assessed the correlation between GC content and sequencing coverage depth. Genome size estimation and depth distribution profiling were performed through 17-mer frequency analysis of high-quality reads, with subsequent statistical characterization of depth uniformity. De novo assembly was conducted via SOAPdenovo2 (v2.04) [19]. PE reads were aligned to preliminary assemblies using SOAPaligner (v2.21) [19] to assess coverage uniformity. Scaffold refinement was performed using GapCloser (v1.12) [20] to resolve ambiguous regions and improve assembly continuity through systematic gap closure.

### 2.4. Genome Annotation

Protein-coding sequences (CDSs) were predicted using an evidence-based consensus approach integrating Glimmer (v3.0.2) [21], GeneMarkS (v4.17) [22], and Prodigal (v2.6.3) [23]. Scaffolds were classified as plasmid-derived sequences using PlasFlow v1.1 (confidence threshold ≥ 0.95) [24]. Functional annotation was performed using five databases: NR (NCBI non-redundant, BLASTp (https://blast.ncbi.nlm.nih.gov/, accessed on 17 November 2025) [25], e-value < 1 × 10^−5^), Swiss-Prot (https://www.ebi.ac.uk/uniprot/, accessed on 17 November 2025) (UniProt curated sequences) [26], Pfam (v32.0; HMMER3 [27], cutoff < 1 × 10^−10^) [28], eggNOG (v5.0) [29], and Gene Ontology (GO; 2022 release, QuickGO) [30]. tRNA and rRNA genes were identified using tRNAscan-SE (v2.0, covariance model) [31] and Barrnap (v0.9) [32], respectively. A linear pseudomolecule representation was generated using CGView Server (v1.0) [33]. To construct a comprehensive functional profile of *Paenarthrobacter* sp. FR1, the predicted genes were assigned to metabolic pathways and orthologous groups using the KEGG (http://www.genome.jp/kegg/, accessed on 17 November 2025) [34] and COG (http://www.ncbi.nlm.nih.gov/COG/, accessed on 17 November 2025) [35] databases.

### 2.5. Phylogeny Analysis

The 16S rDNA sequence of strain FR1 was compared against the EzBioCloud 16S database (Version 20250421) [36] and subjected to phylogenetic analysis using MEGA 12 [37]. A neighbor-joining tree was constructed based on the Tajima-Nei model, with site-specific rate variation modeled by a gamma distribution (shape parameter α = 5). Branch robustness was evaluated by 1000 bootstrap replicates. Genome-wide relatedness across *Paenarthrobacter* strains was assessed through an integrative computational framework combining three analytical metrics: The BLAST-based ANIb algorithm implemented in Pyani (v0.2.10) [38] provided average nucleotide identity (ANI) scores; in silico DNA-DNA hybridization (DDH) values were derived using the Genome-to-Genome Distance Calculator 3.0 (GGDC) [39] with Formula 2 (recommended for draft genomes); and MUMmer (v4.0) [40] enabled comparative profiling of GC content variations through whole-genome alignments.

### 2.6. CAZyme Analysis

CAZyme annotation was performed by submitting the predicted protein repertoire of *Paenarthrobacter* sp. FR1 to the dbCAN3 meta server (v4.2) [41] for analysis using the CAZy database (updated 6 January 2025) [42]. The analysis integrated predictions from HMMER (dbCAN HMM profiles), DIAMOND (CAZy family-specific sequences), and Hotpep (conserved peptide patterns), with positive hits requiring consensus from at least two tools [43]. The identified CAZyme-encoding genes were systematically cataloged and classified into their respective families. To confirm the functional annotation of key enzymes implicated in pectin metabolism, all putative CAZymes were subjected to verification using NCBI BLASTP [25] against the non-redundant (nr) protein database. Conserved domain architectures were further assessed via the NCBI Conserved Domain Database (CDD) [44]. To comprehensively assess the pectin-degrading potential of FR1 and distinguish its unique enzymatic profile from related species, a detailed comparative CAZyme analysis was conducted. The predicted protein sets of three closely related *Paenarthrobacter* strains (*Paenarthrobacter nicotinovorans* DSM 420^T^, *Paenarthrobacter aromaticivorans* MMS21-TAE1-1^T^, *Paenarthrobacter aurescens* NBRC 12136^T^) were retrieved from NCBI and independently annotated for CAZymes using the same dbCAN3 pipeline and stringent criteria. The resulting CAZyme profiles of FR1 and the three reference strains were then compared and tabulated to highlight the relative enrichment or unique presence of specific enzyme families, particularly those involved in pectin metabolism.

### 2.7. Secretion System of Strain FR1

To identify the genetic locus of the secretion system in strain FR1, an in silico analysis of its whole genome sequence was conducted. The genome was scanned for conserved gene clusters encoding known secretion systems using the MacSyFinder (v2.1.2) [45] tool. This program utilizes a library of hidden Markov model (HMM) profiles to detect co-localized genes that constitute the core components of such systems. The putative secretion system gene cluster identified in strain FR1 was further examined, and individual open reading frames (ORFs) were annotated using BLASTp searches against the NCBI non-redundant protein database [46] to confirm their homology to known secretion system components.

### 2.8. Analysis of Transport Proteins and Transmembrane Proteins

The predicted proteome of strain FR1 was systematically analyzed to identify and classify all putative transport proteins. This was accomplished by performing a BLASTp search of each protein sequence against the curated sequence database of the Transporter Classification Database (TCDB) [47]. Hits with an E-value threshold of 1e−5 or lower were considered significant. Proteins showing significant homology were subsequently classified and annotated with a specific Transporter Classification (TC) number, which categorizes transporters based on their class, subclass, family, and substrate specificity. Furthermore, to identify all potential transmembrane proteins within the proteome, the TMHMM Server v. 2.0 [48] was used to predict the presence and topology of transmembrane helices in each protein sequence.

## 3. Results

### 3.1. Description of Paenarthrobacter sp. FR1

Colonies of strain FR1 were creamy after 48 h of growth on marine agar 2216E plates at 28 °C, appearing opaque with smooth surfaces, intact edges, and diameters of approximately 1–2 mm. Gram staining was positive. Growth experiments confirmed the strain’s pectinolytic ability, as it grew in both solid and liquid LMO medium with 0.5% pectin as the sole carbon source (Figure S1). In liquid culture, the maximum OD_600_ reached 0.32 ± 0.04 (*n* = 3) after 5 days of incubation. The growth curve of the strain on pectin (as sole carbon source) from 0 to 22 h is detailed in Supplementary Figure S2. To experimentally validate the pectin-degrading potential predicted by the genomic analysis of FR1, we quantitatively measured the crude pectin lyase (PL) activity in the bacterial culture supernatant following 8 h of cultivation in LMO medium supplemented with 0.5% percent pectin. PL activity was determined spectrophotometrically by measuring the rate of beta-elimination product formation (Delta 4,5-unsaturated oligogalacturonates) at 235 nm. The results demonstrated that the *Paenarthrobacter* sp. FR1 culture supernatant exhibited significant extracellular PL activity. The average specific activity of the crude enzyme solution was determined to be 115.284 ± 9.283 nmol/min/mL (using polygalacturonate as the substrate at 50 °C and pH 9.0). This finding provides functional evidence for the presence of active extracellular pectinolytic enzymes in FR1.

### 3.2. Genome Overview

Based on 16S rRNA gene sequence analysis, strain FR1 was identified as a member of the genus *Paenarthrobacter*, sharing 98.96% sequence similarity with the type strain *Paenarthrobacter aurescens* NBRC 12136^T^. The general genomic and physiological characteristics of strain FR1 are summarized in Table 1.

**Figure 1:**
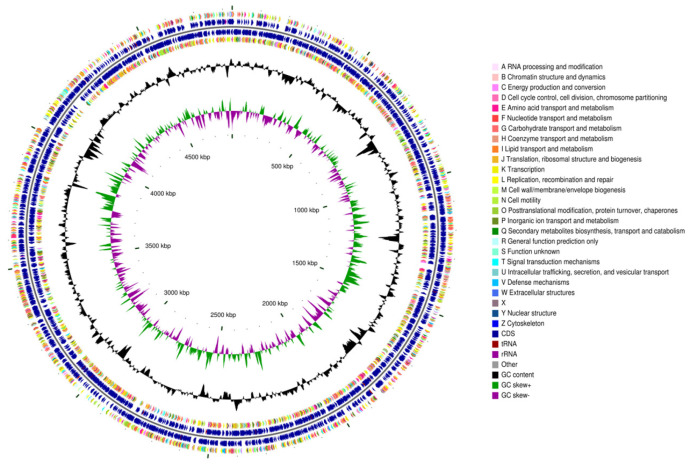
General features of the genome of *Paenarthrobacter* sp. FR1. The CGView genome circle map (the circular visualization is a schematic and does not imply biological completion) of *Paenarthrobacter* sp. FR1, organized from outermost to innermost as follows: Circles 1 and 4 display CDS on the positive and negative strands (color-coded by COG functional categories); circles 2 and 3 show CDS, tRNA, and rRNA for the positive and negative strands, respectively; circle 5 indicates the GC content (outward peaks = GC above genome average, inward peaks = GC below average, with peak height reflecting deviation magnitude); circle 6 displays GC-skew values.

The draft genome assembly comprised 41 contigs (contig N50 = 335,934 bp; largest contig = 1,018,001 bp) totaling 4,834,017 bp with a 62.92% GC content (Figure 1). Assembly completeness (99.77%) and contamination (0%, with no heterogeneous markers detected) were assessed using CheckM v1.2.3 with the Micrococcaceae lineage-specific marker gene set [49] and was based on genes annotated by the Prokaryotic Genome Annotation Pipeline (PGAP) [50].

**Figure 2:**
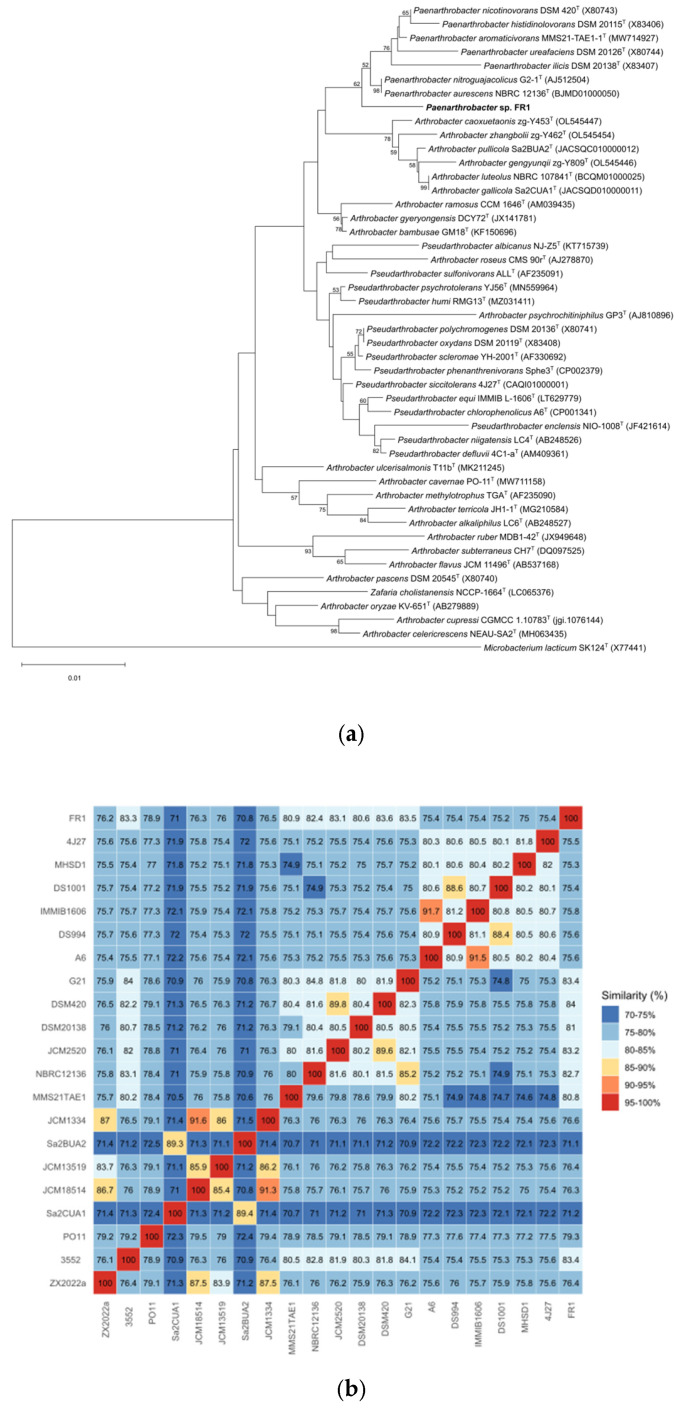
Phylogenetic analyses of *Paenarthrobacter* sp. FR1. (**a**)**:** The phylogenetic tree of *Paenarthrobacter* sp. FR1 based on 16S rRNA gene sequences. The outgroup is *Microbacterium lacticum* SK124^T^ (X77441). The tree was created using MEGA 12. (**b**)**:** ANI value (%) heatmap for 21 bacterial strains, calculated using BLASTn alignment. ZX2022a, *Arthrobacter alkaliphilus* ZX 2022a^T^; 3552, *Arthrobacter bambusae* 3552^T^; PO11, *Arthrobacter cavernae* PO-11^T^; Sa2CUA1, *Arthrobacter gallicola* Sa2CUA1^T^; JCM18514, *Arthrobacter gyeryongensis* JCM 18514^T^; JCM13519, *Arthrobacter methylotrophus* JCM 13519^T^; Sa2BUA2, *Arthrobacter pullicola* Sa2BUA2^T^; JCM1334, *Arthrobacter ramosus* JCM 1334^T^; MMS21TAE1, *Paenarthrobacter aromaticivorans* MMS21-TAE1-1^T^; NBRC12136, *Paenarthrobacter aurescens* NBRC 12136^T^; JCM2520, *Paenarthrobacter histidinolovorans* JCM 2520^T^; DSM20138, *Paenarthrobacter ilicis* DSM 20138; DSM420^T^, *Paenarthrobacter nicotinovorans* DSM 420^T^; G21, *Paenarthrobacter nitroguajacolicus* G2-1^T^; A6, *Pseudarthrobacter chlorophenolicus* A6^T^; DS994, *Pseudarthrobacter defluvii* DS994^T^; IMMIB1606, *Pseudarthrobacter equi* IMMIB L-1606^T^; DS1001, *Pseudarthrobacter niigatensis* DS1001^T^; MHSD1, *Pseudarthrobacter phenanthrenivorans* MHSD1^T^; 4J27, *Pseudarthrobacter siccitolerans* 4J27^T^; FR1, *Paenarthrobacter* sp. FR1 (our strain).

Figure 2 shows the 16S rRNA-based phylogenetic tree and ANI values (%) of the 21 strains. *Paenarthrobacter* sp. FR1 shared the highest average nucleotide identity (ANI) of 83.56% and digital DNA-DNA hybridization (dDDH) value of 27.8% (confidence interval 25.5–30.3%) with its closest type strain *Paenarthrobacter nicotinovorans* DSM 420^T^. These values fall below the standard species demarcation thresholds (ANI < 95%, dDDH < 70%) [51]. The genomic G + C content differed by 0.18% from that of its nearest type strain. Phylogenomic analysis and polyphasic taxonomic evidence indicate strain FR1 represents a distinct novel species within the genus *Paenarthrobacter*. Collective data support strain FR1 as a potential novel strain constituting an independent species in *Paenarthrobacter*.

**Figure 3:**
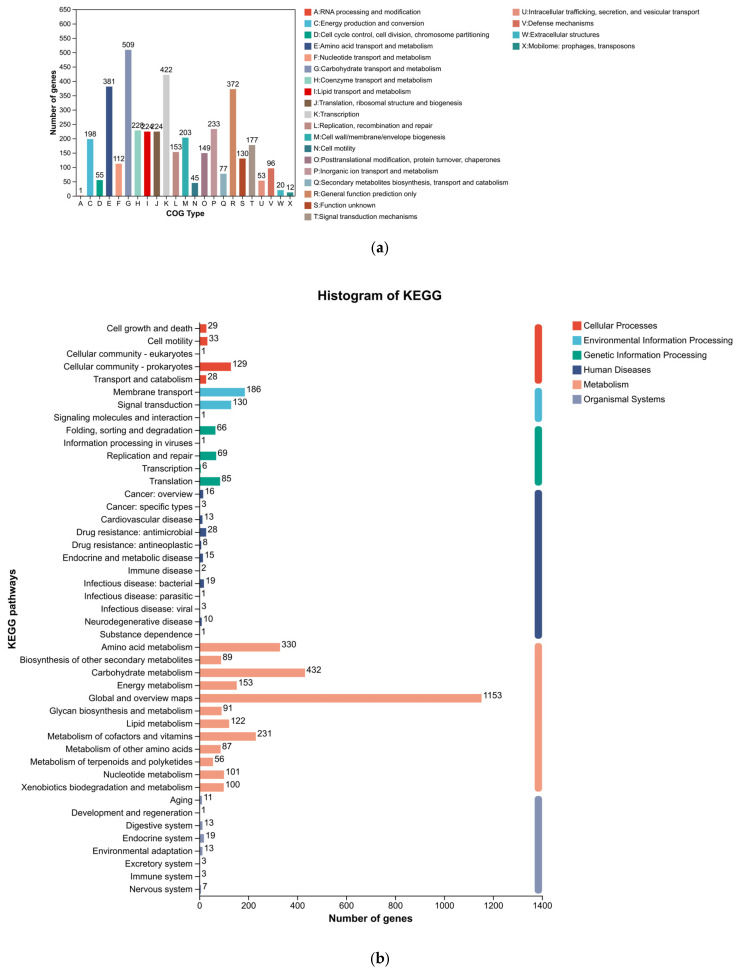

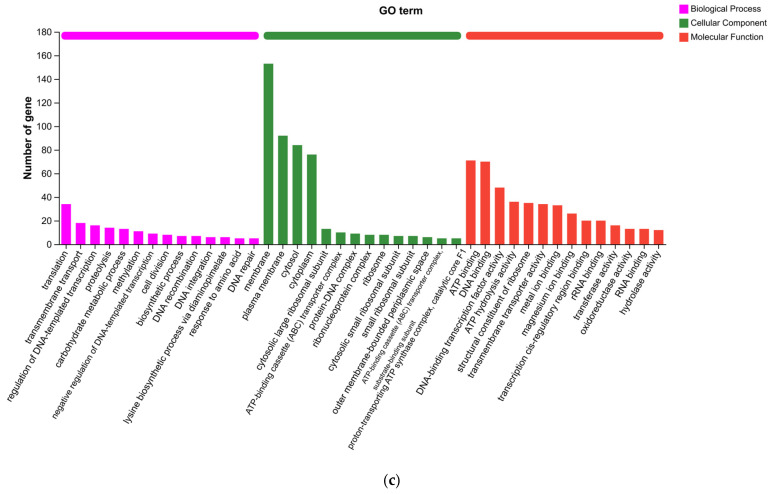
Functional annotation of the *Paenarthrobacter* sp. FR1 genome. (**a**) COG (Clusters of Orthologous Groups) functional classification. (**b**) KEGG (Kyoto Encyclopedia of Genes and Genomes) pathway annotation. (**c**) Gene Ontology (GO) classification by Biological Process, Cellular Component, and Molecular Function.

Functional annotation using the COG, KEGG, and GO databases provided a holistic view of the metabolic and cellular processes of *Paenarthrobacter* sp. FR1 (Figure 3). A significant portion of the genome was dedicated to nutrient processing, with “Carbohydrate transport and metabolism” representing the largest functional category in the COG analysis. This finding is consistent with the KEGG pathway mapping, which revealed extensive networks for carbohydrate metabolism. The GO analysis further detailed these functions across biological processes, cellular components, and molecular activities.

### 3.3. CAZyme Profile and Pectin Degradation Pathway

**Figure 4:**
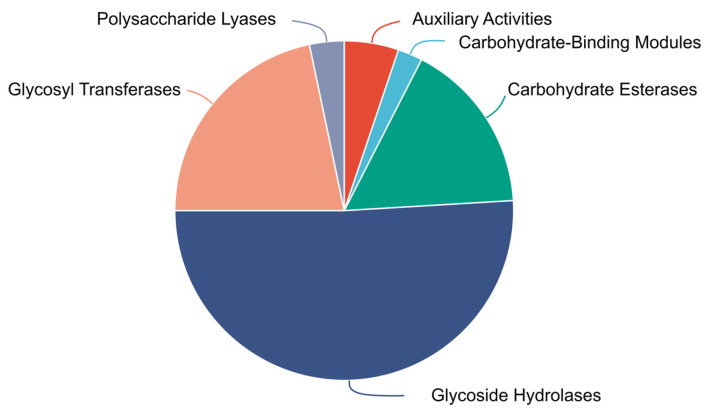
Proportional distribution of Carbohydrate-Active enZyme (CAZyme) families identified in the genome of *Paenarthrobacter* sp. FR1.

Strain FR1 exhibited an ability to grow using pectin as a single carbon source (Figure S1). Analysis of the *Paenarthrobacter* sp. FR1 genome revealed that it encodes a diverse repertoire of CAZymes (Figure 4, Table S1), comprising 108 glycoside hydrolases (GHs), 46 glycosyltransferases (GTs), 7 polysaccharide lyases (PLs), 35 carbohydrate esterases (CEs), 5 carbohydrate-binding modules (CBMs), and 11 auxiliary activities enzymes (AAs). Functional predictions for these enzymes are based on the protein models derived from genomic annotation. Crucially, a preliminary comparative analysis of the CAZyme profiles between FR1 and three closely related *Paenarthrobacter* strains was performed. While the limited number of available strains for this genus restricts the ability to claim statistically significant enrichment, this simple comparison suggested a trend toward a higher count of polysaccharide-degrading enzymes in FR1, specifically demonstrating that the total gene numbers for Glycoside Hydrolases (GH) and Carbohydrate Esterases (CE) families were higher in FR1 than in its relatives. This observation indicates that FR1 may possess an enhanced genetic potential for complex carbohydrate utilization compared to its nearest relatives. A detailed breakdown of the CAZyme families across all four genomes is provided in Supplementary Table S2.

Pectin, primarily composed of homogalacturonan (HG) and rhamnogalacturonan (RG) domains [1,2], undergoes a stepwise degradation process initiated by esterase-mediated de-esterification, followed by backbone cleavage, side chain modification, and oxidative assistance, culminating in oligosaccharide transport and catabolism [52]. Based on genomic homology, strain FR1 is proposed to initiate pectin degradation through a CE8 pectin methylesterase (protein ID: WP_434372502.1), specifically hydrolyzing methyl ester groups of the homogalacturonan backbone to produce low-methoxyl pectin and methanol [53,54]; concurrently, CE12 rhamnogalacturonan acetylesterase (protein ID: WP_434371323.1) targets acetyl modifications in rhamnogalacturonan-I regions to expose glycosidic bonds—a preliminary de-esterification step that constitutes the rate-limiting step [55].

Subsequent backbone depolymerization relies on synergistic hydrolase-lyase actions where homogalacturonan domains PL1 pectin lyase (protein ID: WP_434372503.1) preferentially cleaves highly methyl-esterified HG with ≥70% esterification to generate Δ4:5 unsaturated oligosaccharides [56]; PL11 rhamnogalacturonan lyase (protein ID: WP_434374095.1) acts on de-esterified RG regions [57]; and GH28 endorhamnogalacturonase (protein IDs: WP_144647454.1, WP_434373709.1) cuts α-1,4-galacturonidic linkages to form oligogalacturonides [58].

Concurrently, RG-I backbone hydrolysis is dominated by GH43 α-L-arabinofuranosidase (protein IDs: WP_144652631.1, WP_434371581.1, WP_434374669.1) and GH2 β-galactosidase (protein IDs: WP_434371168.1, WP_434372917.1, WP_434374638.1) [59,60], with GH78 α-L-rhamnosidase (protein IDs: WP_434371998.1, WP_434371982.1, WP_434371327.1) removing RG-I rhamnose side chains to enhance backbone accessibility [61]. Side chains are further processed by GH51 α-L-arabinofuranosidase (protein IDs: WP_434372529.1, WP_434373312.1), eliminating arabinan steric hindrance, and GH35 β-galactosidase (protein IDs: WP_434371077.1, WP_434373890.1, WP_434374630.1) cleaving β-1,4-galactan branches [62].

**Figure 5:**
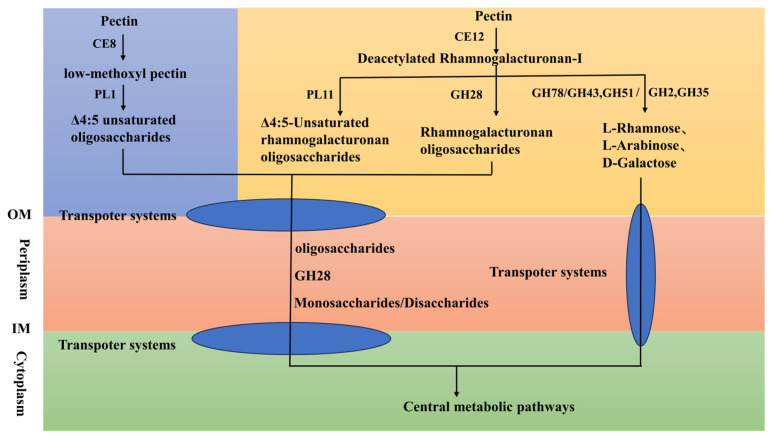
Pectin degradation pathways in *Paenarthrobacter* sp. FR1. Abbreviations: IM, inner membrane; OM, outer membrane; CE8, pectin methylesterase, CE12, rhamnogalacturonan acetylesterase, PL1, pectin lyase, PL11, rhamnogalacturonan lyase, GH28, endorhamnogalacturonase, GH78, α-L-rhamnosidase, GH43, α-L-arabinofuranosidase, GH2, β-galactosidase, GH51, α-L-arabinofuranosidase, GH35, β-galactosidase.

During protopectin dissociation GH78 enzyme complexes (protein IDs: WP_434371998.1, WP_434371982.1, WP_434371327.1) disrupt rhamnose-xyloglucan linkages at pectin-hemicellulose interfaces [63], cooperating with AA7 Cellooligosaccharide dehydrogenase (protein IDs: WP_144652103.1, WP_434372555.1, WP_434373944.1), which oxidizes C6-hydroxyl groups of galactose/arabinose residues to generate chain-scission-inducing reactive aldehydes [64], while GH35 β-galactosidase (protein IDs: WP_434371077.1, WP_434373890.1, WP_434374630.1) cleaves β-1,4-galactan side-chains, collectively enabling efficient cell wall-bound pectin release [63]. Figure 5 illustrates the possible pathways through which the FR1 strain degrades pectin.

### 3.4. Protein Secretion Systems Enable Extracellular Enzymatic Activity

A detailed genomic search revealed that *Paenarthrobacter* sp. FR1 possesses comprehensive genetic machinery for protein export, which is essential for its extracellular activities, including pectin degradation [65]. Two complete and distinct protein translocation systems, the general secretory (Sec) and the twin-arginine translocation (Tat) pathways, were identified (Figure S2).

The Sec-SRP system, which is responsible for the transport of most proteins in an unfolded state [66], was found to be fully intact. This includes the genes encoding the core membrane channel components *secY*, *secE*, and *secG*, and the crucial ATPase motor *secA*. Furthermore, the presence of the signal recognition particle pathway genes (*ffh*, *ftsY*) and various genes for accessory proteins (*secD*, *secF*, *yajC*, *yidC*) suggests a highly efficient and robust apparatus for secreting proteins. This system is the presumed primary pathway for the export of the bulk of pectinolytic enzymes, such as polygalacturonases and pectin lyases, which act on the pectin polymer outside the cell.

Complementing the Sec pathway, a complete Tat system, comprising genes *tatA, tatB*, and *tatC*, was also identified. The Tat pathway is specialized in translocating fully folded proteins, which is critical for enzymes that require cytoplasmic assembly or the insertion of cofactors prior to export [67]. The existence of this parallel pathway suggests that FR1 may secrete a more diverse and complex set of enzymes, potentially including specialized pectinases or other proteins that could contribute to its ability to thrive in a plant-associated environment. Together, the presence of these two complete protein secretion gene systems provides a strong genetic foundation for FR1’s ability to export a wide arsenal of enzymes, enabling the effective extracellular breakdown of complex polysaccharides like pectin.

### 3.5. Extensive Network of Transporters for Nutrient Uptake

To complement its powerful extracellular enzymatic capability, the genome of *Paenarthrobacter* sp. FR1 encodes an extensive repertoire of transmembrane and transport proteins, indicating a profound adaptation for scavenging nutrients from its environment.

A genome-wide analysis identified a total of 1087 proteins containing at least one transmembrane helix (Table S3), 809 of which were specifically classified as transport proteins based on annotation using the Transporter Classification Database (TCDB) (Table S4). This large number of transporters signifies a robust capacity for shuttling a wide variety of substrates across the cell membrane.

In the context of pectin degradation, this transport network is fundamentally important. While the previously described secretion systems are responsible for the external depolymerization of pectin, the resulting soluble mono- and oligosaccharides (such as D-galacturonic acid, rhamnose, arabinose, and galactose) must be efficiently imported into the cell for catabolism. It is therefore highly probable that a significant fraction of these 809 transporters are dedicated to the uptake of these pectin-derived sugars. These likely include members of major transporter families such as the ATP-binding cassette (ABC) superfamily and the Major Facilitator Superfamily (MFS), which are commonly involved in carbohydrate transport in bacteria [68,69,70].

The abundance and diversity of these transporters underscore a genomic specialization for its lifestyle. This extensive transport system ensures that the breakdown products generated by extracellular enzymes are rapidly captured and utilized, allowing FR1 to effectively capitalize on the availability of complex plant biomass.

## 4. Discussion

This study elucidates the genetic basis underlying the pectin-degrading ability of *Paenarthrobacter* sp. FR1, a potentially novel strain isolated from East China Sea intertidal sediment. Genomic analysis revealed that FR1 possesses a sophisticated, multi-enzyme system capable of disrupting key barriers to pectin recalcitrance, including methyl and acetate esterification as well as arabinan crosslinking. The synergistic action between carbohydrate esterases (e.g., CE8, CE12) and backbone-degrading enzymes (e.g., GH28 hydrolases, PL1 lyases) facilitates a stepwise deconstruction of pectin polymers.

Furthermore, the presence of AA7 auxiliary activity enzymes suggests a mechanism for cleaving phenolic acid crosslinks, which could mitigate the physical shielding effect of lignin on pectin and enhance the overall degradation efficiency [71]. A particularly notable feature of FR1 is its innate ability to simultaneously target both the homogalacturonan (HG) and rhamnogalacturonan (RG) structural domains of pectin, a strategic advantage that likely significantly enhances carbon bioavailability from complex pectin substrates.

The genomic evidence, coupled with experimental validation of its growth on pectin as a sole carbon source, suggests that FR1 may serve as a key player in the breakdown of plant biomass in its native marine environment. The release and degradation of pectin from terrestrial plant debris are critical steps in the marine carbon cycle [71], and strains with such dedicated enzymatic machinery, like FR1, contribute significantly to our understanding of the fate of this biomass in coastal ecosystems.

From a biotechnological perspective, the enzymatic arsenal of *Paenarthrobacter* sp. FR1 presents significant potential. Its versatility in attacking diverse pectin structures, owing to complementary hydrolytic and lytic systems, makes it a highly promising candidate for industrial applications. Beyond pectinases, the co-occurrence of other plant biomass-degrading enzymes, such as GH5 cellulases and AA3 oxidoreductases [72,73], indicates an innate capacity for the synergistic degradation of complex plant polymers. This comprehensive enzymatic profile suggests that strain FR1, or its enzyme cocktails, could be effectively harnessed for biofuel production, agricultural waste valorization, and the development of specialized processing aids for the pectin-rich fruit and vegetable industry [9,74].

While our genomic analysis provides a robust blueprint for FR1’s pectinolytic system, future studies should focus on the biochemical characterization of key enzymes to validate their predicted functions and explore their catalytic efficiencies. Furthermore, transcriptomic and proteomic analyses could reveal how this enzymatic arsenal is regulated in response to pectin, paving the way for optimizing this strain for industrial applications.

## 5. Conclusions

In this study, we successfully isolated and characterized a novel marine bacterium, *Paenarthrobacter* sp. FR1, providing the first definitive evidence of pectin-degrading capabilities within the genus *Paenarthrobacter*. Through comprehensive genomic analysis, we revealed that strain FR1 possesses a sophisticated and diverse arsenal of Carbohydrate-Active Enzymes (CAZymes) that enable the complete deconstruction of complex pectin polymers. The genomic data elucidated a multi-step degradation strategy involving both hydrolytic and oxidative pathways that effectively target the primary structural domains of pectin.

Phylogenomic analysis strongly supports the classification of FR1 as a potential new species, significantly expanding the known metabolic repertoire and ecological functions of this genus beyond pollutant degradation. Our findings position *Paenarthrobacter* sp. FR1 as a previously unrecognized player in the marine carbon cycle, contributing to the breakdown of plant biomass in coastal ecosystems. Furthermore, the unique enzymatic machinery identified in this strain represents a valuable resource with significant biotechnological potential for industrial applications, including biofuel production, food processing, and the valorization of pectin-rich agricultural waste.

## Figures and Tables

**Table 1:** General genomic features and physiological characteristics of *Paenarthrobacter* sp. FR1 according to MIGS recommendations.

Item	Description
Classification	Domain Bacteria
	Phylum Actinomycetota
	Class Actinomycetes
	Order Micrococcales
	Family Micrococcaceae
	Genus *Paenarthrobacter*
General features	
Gram stain	positive
Cell shape	Rod
Colony color	white
Oxygen requirements	Aerobic
Motility	Motile
MIGS data	
BioProject	PRJNA1277493
BioSample	SAMN49110650
Investigation type	Bacteria
Project name	*Paenarthrobacter* sp. FR1 Genome sequencing
Latitude and longitude	31°26′ N, 121°57′ E
Depth	−0.5 m
Geographical location	Chongming Dongtan, Shanghai
Collection date	December 2024
Environment (material)	Marine sediment [ENVO:03000033]
Environment (feature)	Marine sediment [ENVO:03000033]
Environment (package)	Sediment [ENVO:00002007]
Isolation and growth condition	2216 broth
Sequencing method	Illumina PE150
Finishing quality	High-Quality Draft Genome
Fold coverage	262×
Assembly method	SOAPdenovo2 (v2.04)
Genomic features	
Size (bp)	4,834,017
G + C content (%)	62.92
Number of predicted CDSs	4498
Genes assigned to eggNOG	3638
Genes assigned to KEGG	3224
Number of rRNA genes	4
Number of tRNAs	54

## Data Availability

The whole genome sequence of *Paenarthrobacter* sp. strain FR1 has been deposited in the GenBank database under the WGS project JBPFSF01 (BioProject: PRJNA1277493, BioSample: SAMN49110650). The strain FR1 has been submitted to the Marine Culture Collection of China (MCCC; http://www.mccc.org.cn/) under accession number MCCC 1K10014.

## Supplementary Materials

The following supporting information can be downloaded at: https://www.mdpi.com/article/10.3390/microorganisms14010039/s1, Table S1: Pectin-Degradation-Related CAZyme Enzymatic System of *Paenarthrobacter* sp. FR1; Figure S1: Growth of strain FR1 on LMO medium with 0.5% pectin as the sole carbon source. a: Liquid medium, b: Solid medium; Figure S2: Growth curve of the strain in liquid LMO medium using pectin as the sole carbon source from 0 to 22 h. Data are presented as the mean optical density (OD600) of three biological replicates (*n *= 3); Figure S3: A model of the protein export machinery in *Paenarthrobacter* sp. FR1 based on genomic analysis. The complete general secretory (Sec) and twin-arginine translocation (Tat) pathways are shown. Components present in the genome of strain FR1 are indicated in red; Table S2: Enumeration of CAZyme family genes in strain FR1 and three closely related *Paenarthrobacter* strains; Table S3: Comprehensive list of proteins containing one or more transmembrane helices identified in *Paenarthrobacter* sp. FR1; Table S4: A complete list of the 809 predicted transport proteins in *Paenarthrobacter* sp. FR1, with classifications from the Transporter Classification Database (TCDB).
