# Supplementary material for: Genomic Analysis of Paenarthrobacter sp. FR1 Reveals Its Marine-Adapted Pectin-Degrading System and Ecological Role in Carbon Cycling
Source: Microorganisms. 2025 Dec 23;14(1):39. doi: 10.3390/microorganisms14010039 (PMC12844168; doi:10.3390/microorganisms14010039)
Supplement: Supplementary file 1 [file microorganisms-14-00039-s001.zip › figure_S3_SECRETION.pdf]

BACTERIAL SECRETION SYSTEM

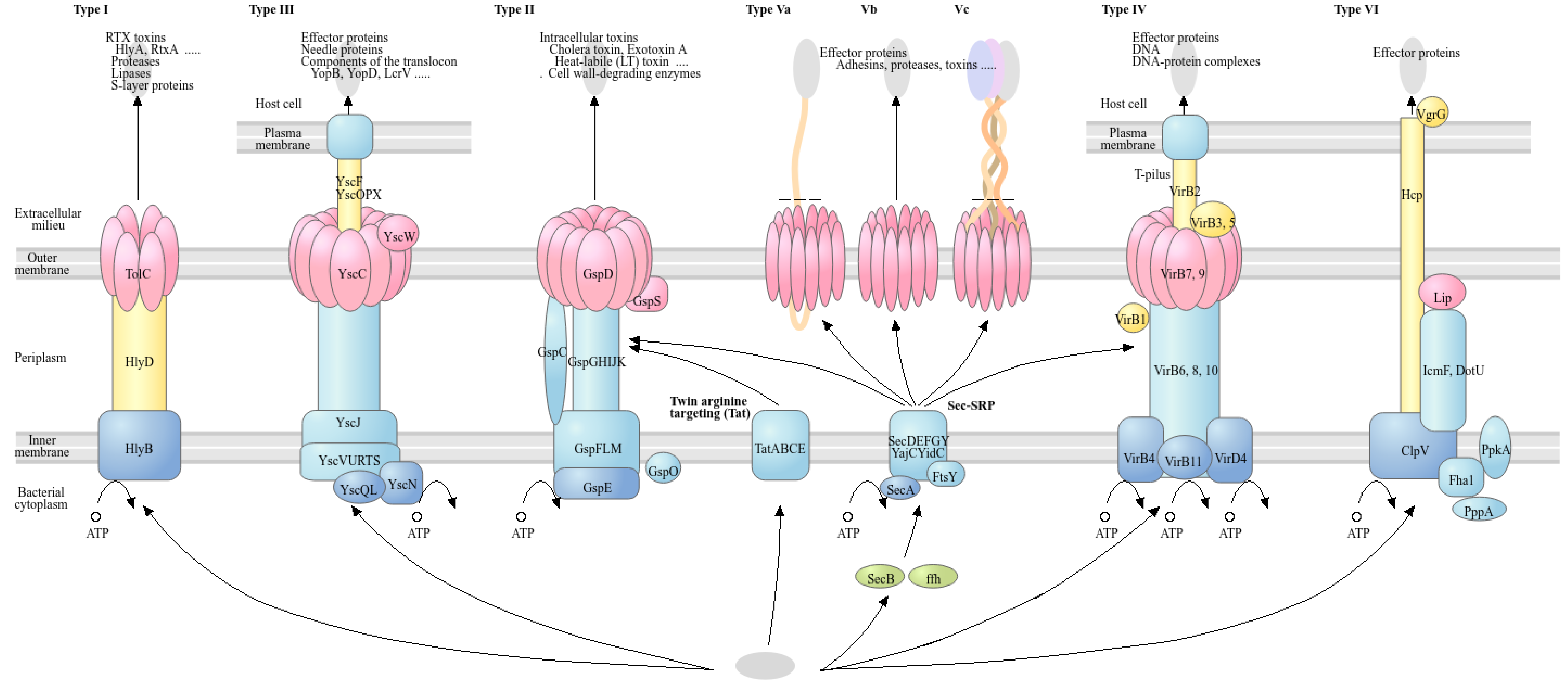

Type I

Outer membrane protein (OMP)	TolC
Membrane fusion protein	HlyD
ABC transporter	HlyB

Type III

Needle	YscF		
	YscO	YscP	YscX
Secretin	YscC		
OMP	YscW		
Inner membrane protein (IMP)	YscJ	YscR	YscS
	YscT	YscU	YscV
ATPase	YscN		
ATPase-associated protein	YscQ	YscL	

Type II

Secretin	GspD		
OMP	GspS		
IMP	GspC	GspF	GspG
	GspH	GspI	GspJ
	GspK	GspL	GspM
ATPase	GspE		
Leaderpeptidase	GspO		

Type Va **Vb** **Vc**

	Va	Vb	Vc
OMP	VacA	ShlB	YadA
		ShlA	YadB/C

Type IV

Periplasmic protein	VirB1		
Surface/pilus protein	VirB2	VirB3	VirB5
OMP	VirB7	VirB9	
IMP	VirB6	VirB8	VirB10
ATPase	VirB4	VirB11	VirD4

Type VI

Secreted substrate	VgrG		
	Hcp		
OMP	Lip		
IMP	IcmF	DotU	
ATPase	ClpV		
Regulatory protein	PpkA	Fha1	PppA

Sec-SRP

IMP	SecD/F	SecE	SecG
	SecY	YajC	YidC
ATPase	SecA		
Secretion monitor	SecM		
SRP receptor	FtsY		
Targeting protein	SecB	ffh	

Twin arginine targeting (Tat)

IMP	TatA	TatB	TatC	TatE

03070 9/10/20

(c) Kanehisa Laboratories
