# Supplementary figures and images for: Genomic Analysis of Paenarthrobacter sp. FR1 Reveals Its Marine-Adapted Pectin-Degrading System and Ecological Role in Carbon Cycling
Source: Microorganisms. 2025 Dec 23;14(1):39. doi: 10.3390/microorganisms14010039 (PMC12844168; doi:10.3390/microorganisms14010039)

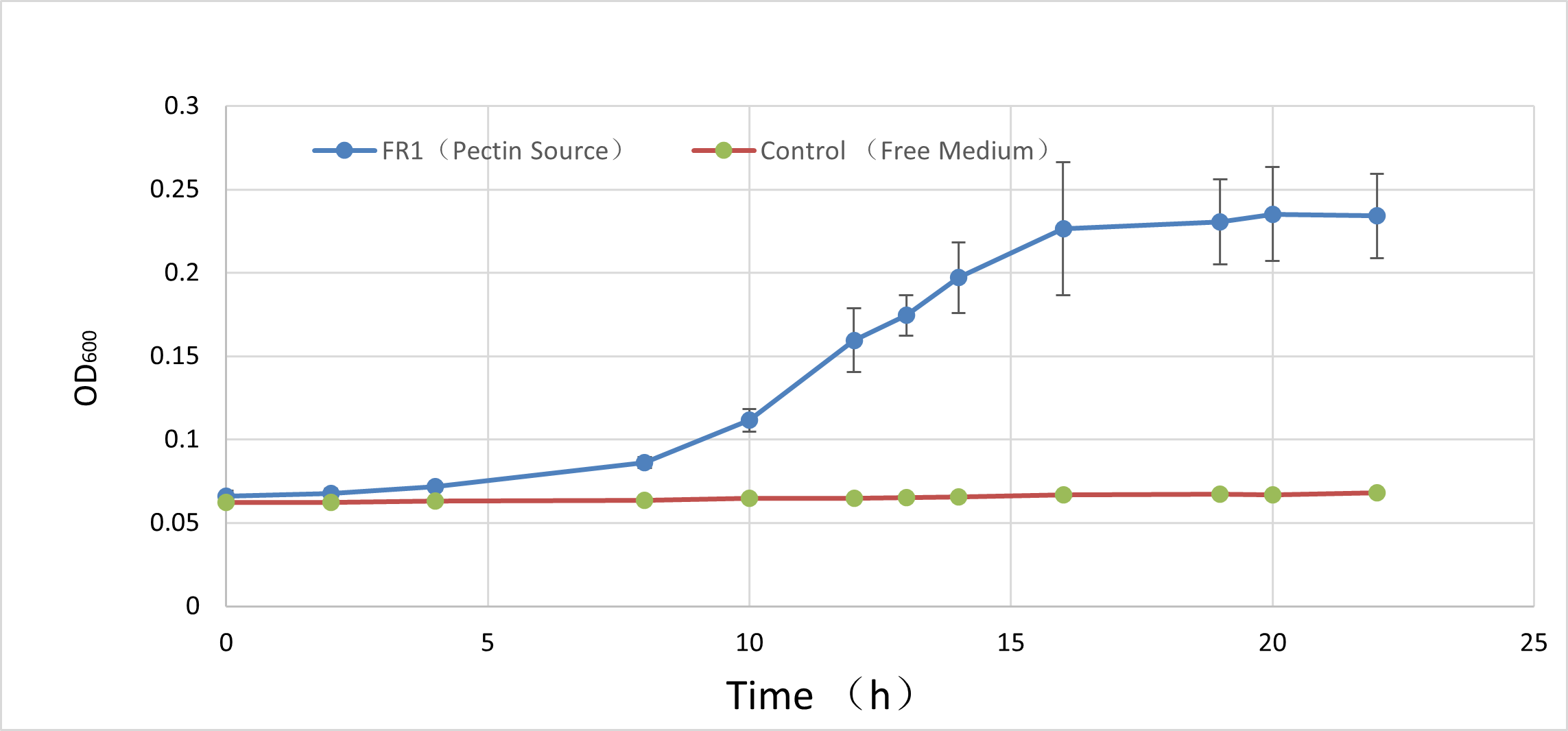

Supplement: Supplementary file 1 [file microorganisms-14-00039-s001.zip › figure_s2_GrowthCurve.tif]

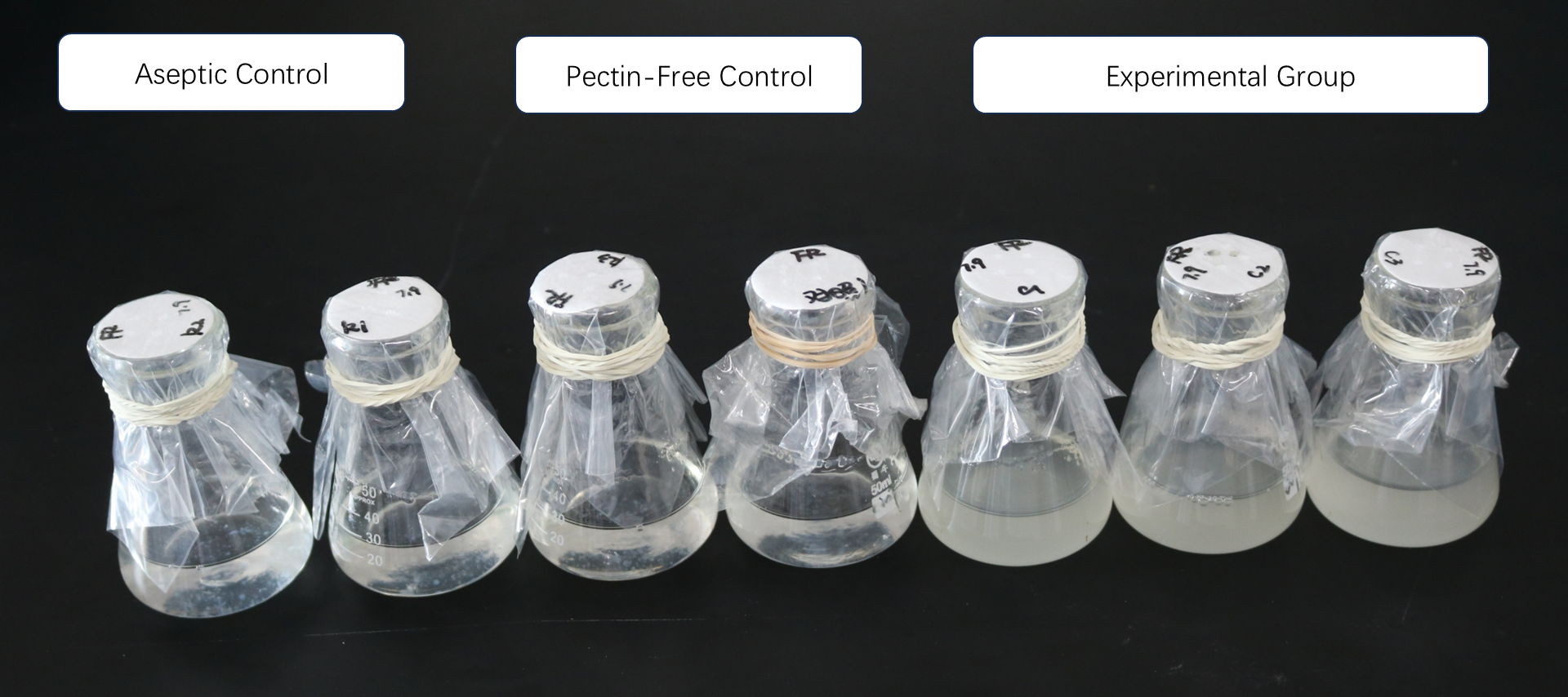

Supplement: Supplementary file 1 [file microorganisms-14-00039-s001.zip › figure_s1a_liquid.png]

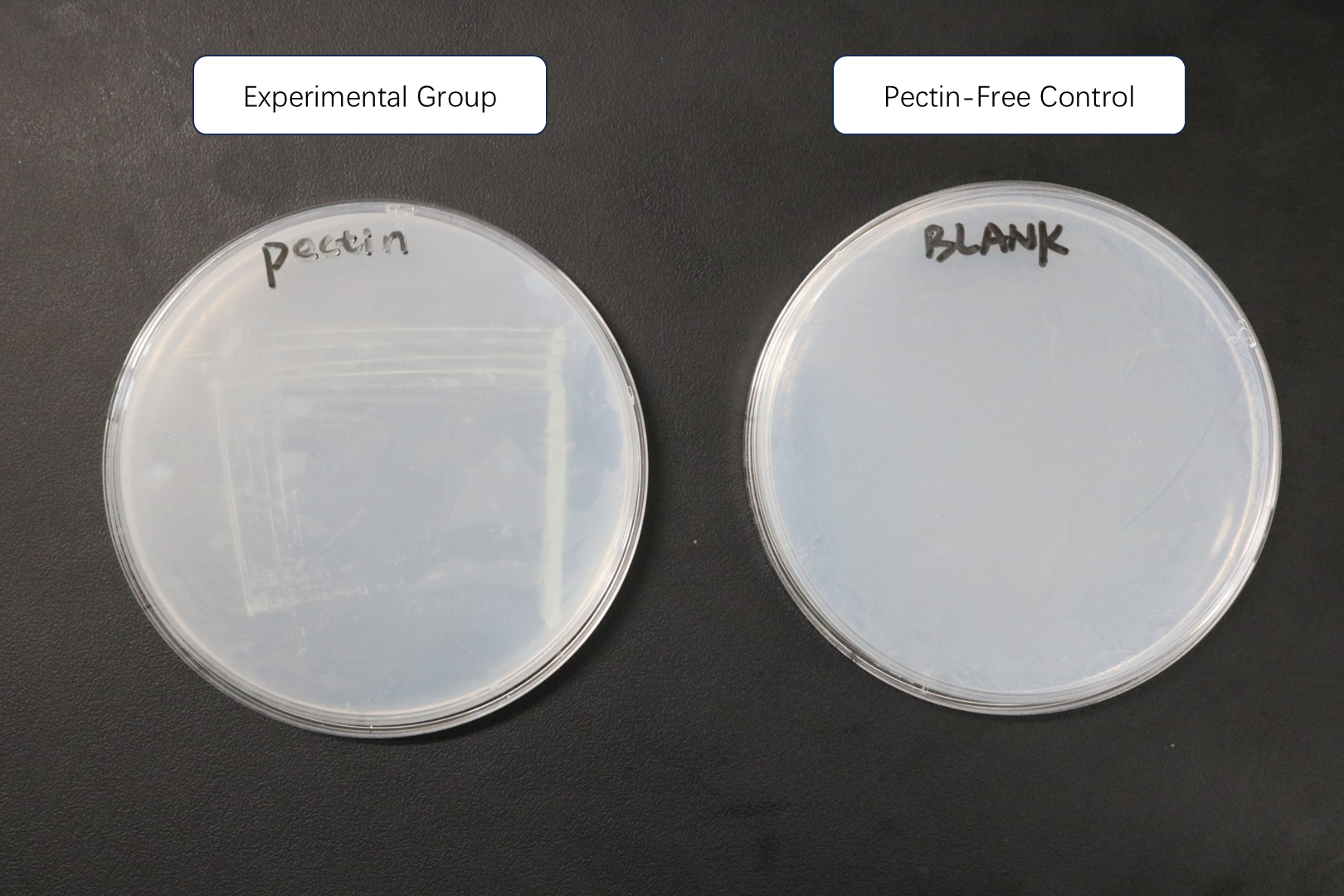

Supplement: Supplementary file 1 [file microorganisms-14-00039-s001.zip › figure_s1b_solid.png]
